# Nephrology's Onboarding Challenge May Reflect a Workforce Challenge

**DOI:** 10.34067/KID.0000001386

**Published:** 2026-09-24

**Authors:** Juliano Alhaddad, Georges Nakhoul

**Affiliations:** Cleveland Clinic Foundation, Cleveland, Ohio

**Keywords:** medical education

The first year of fellowship represents one of the most challenging transitions in postgraduate medical training. Although the transition from medical school to residency has received substantial attention, the transition from residency to subspecialty fellowship may be similarly stressful, particularly given increased clinical responsibility; greater autonomy; and the absence of standardized approaches to onboarding, orientation, and early-fellowship education. In contrast to residency, where structured orientation and boot camp curricula are increasingly common, practices surrounding the transition to fellowship remain heterogeneous.^1^ This transition may be particularly challenging in nephrology, where fellows are entrusted with the care of medically complex patients with the highest burdens of comorbid disease, medication use, mortality, and long-term care utilization across medical specialties.^2^ These clinical demands may further magnify the challenges of adapting to fellowship-level expectations.

In their survey of 339 adult nephrology fellows, La *et al*. sought to address two important questions.^3^ First, they evaluated fellows' self-perceived preparedness at the start of nephrology fellowship. Second, they examined fellows' experiences with early-fellowship curricula and whether participation in such curricula was associated with perceived preparedness. The authors also explored fellows' preferences regarding educational content and teaching methods that should shape an ideal early-fellowship curriculum.

It is reassuring that nearly three quarters of respondents reported feeling at least moderately prepared when they began nephrology fellowship. However, participation in an early-fellowship curriculum was not associated with greater self-perceived preparedness. In addition to heterogeneity in the content, timing, and participation of early-fellowship curricula, a major potential contributor to this discordance, and to the lack of association with fellows' self-perceived preparedness, is that subjectivity influences both the intervention itself and the outcome being measured. Designing a curriculum or boot camp primarily around learner preferences is inherently limited by the fact that trainees may not yet know what they do not know. Moreover, perceived learning needs are likely to evolve with increasing clinical exposure. A new fellow with limited prior nephrology experience may understandably prioritize the management of urgent electrolyte abnormalities, dialysis-requiring AKI, and basic clinical workflow, as reflected in the current survey. With increasing exposure to longitudinal nephrology care, however, priorities may shift toward home dialysis modalities, chronic dialysis management, glomerular diseases, and other topics requiring greater clinical nuance.^4^

Subjectivity also affects the outcome measure itself. Previous studies have demonstrated poor correlations between self-assessment and objective performance, a discrepancy that may be particularly pronounced during major transitions in training.^5^ Some trainees may underestimate their capabilities, a phenomenon frequently discussed in the context of imposter syndrome, whereas others may overestimate their proficiency, consistent with the Dunning–Kruger effect.^6^ One potential approach to overcoming these limitations is competency-based assessment using entrustable professional activities—units of professional practice that can be entrusted to trainees once sufficient competence has been demonstrated. For example, the University of New Mexico nephrology fellowship recently developed 22 entrustable professional activities spanning ten core nephrology domains and mapped to 38 Accreditation Council for Graduate Medical Education subcompetencies.^7^ Such frameworks could shift assessment away from self-reported preparedness toward longitudinal demonstration of clinical competence.

Yet, even the most rigorous assessment framework cannot substitute for insufficient exposure to nephrology during residency. Indeed, the only factor associated with greater self-perceived preparedness in the study was at least 3 weeks of nephrology exposure during residency. What is more alarming, however, is that fellows who felt unprepared were nearly three times as likely to have completed 2 weeks or less of nephrology rotations in residency as those who felt prepared (17% versus 6%), meaning that some fellows may have entered nephrology fellowship with little or even no prior exposure.

Looking beyond the limitations of self-perceived preparedness and recognizing that perceptions themselves can influence career choices, these findings are concerning at a time when both exposure to and interest in nephrology are declining among residents. The proportion of nephrology fellowship positions filled has fallen substantially over the past 15 years, from 94.1% in 2010 to 65.8% in 2024.^8^ Besides concerns regarding compensation and work–life balance, declining interest in nephrology has been attributed in part to limited exposure and the perceived complexity of the specialty. For example, a recent single-center survey of internal medicine residents found that residents had completed a mean of 1.2 weeks of nephrology-specific rotations across medical school and residency combined. This relationship between exposure and interest is not merely inferential because residents who had seriously considered a career in nephrology had completed nearly three times as many weeks of nephrology rotations as their counterparts (2.3 versus 0.8 weeks, *P* = 0.006).^9^ In addition, even when nephrology exposure does occur, it takes place predominantly during the second and third years of residency, well after medical school and intern year, when more than half of residents have already decided on a fellowship.^10^

Furthermore, exposure to nephrology may be limited not only clinically but also in the scholarly and academic setting. Indeed, and despite a major paradigm shift in guideline-directed medical therapy for CKD, along with important therapeutic advances in GN, nephrology, along with urology, represents only approximately 6% of the American Board of Internal Medicine certification examination blueprint, compared with 9% for several other subspecialties, including endocrinology, infectious diseases, pulmonology, and rheumatology, and 14% for cardiovascular medicine and hematology-oncology combined.^11^

If the results of this survey are generalizable, and if limited exposure contributes to poorer preparation among incoming fellows, then the lack of preparedness may reflect a broader lack of exposure, which in turn may contribute to the current nephrology workforce challenges. These findings should therefore serve as a call to action for the nephrology community to improve exposure to our rapidly evolving and complex field among internal medicine residents. Nephrology faculty are often involved in internal medicine residency program leadership. Although the Accreditation Council for Graduate Medical Education does not require a dedicated core rotation in any subspecialty during internal medicine residency, we should work toward developing both mandatory inpatient and outpatient nephrology rotations that expose residents to the full spectrum of kidney disease while simultaneously attracting future applicants. These rotations should ideally provide multiple weeks of exposure and, when possible, begin during intern year while residents are still considering fellowship and career choices.

With the growing number of patients living with CKD, kidney failure, and kidney transplantation, together with major advances in the treatment of glomerular diseases, the complexity and beauty of nephrology are undoubtedly here to stay. The time has come to take meaningful action to reverse the current decline in interest, particularly when it stems from limited exposure to, and understanding of, the breadth and appeal of the field, and to preserve the future of our specialty.

## Supplementary Material
