# Supplementary material for: Nephrology's Onboarding Challenge May Reflect a Workforce Challenge
Source: Kidney360. 2026 Sep 24;7(9):1928–9. doi: 10.34067/KID.0000001386 (PMC13641447; doi:10.34067/KID.0000001386)
Supplement: Supplementary file 1 [file kidney360-7-1928-s001.pdf]

ASN American Society of Nephrology | JASN Journal of the American Society of Nephrology | KIDNEY360 Accessing Our World From Every Angle | CJASN Clinical Journal of the American Society of Nephrology

ASN Journal Disclosure Form

As per ASN journal policy, I have disclosed any financial relationships or commitments I have held in the past 36 months as included below. I have listed my Current Employer below to indicate there is a relationship requiring disclosure. If no relationship exists, my Current Employer is not listed.

J. Alhaddad reports the following:
Employer: Cleveland Clinic Foundation; Boston Medical Center Health System

I understand that the information above will be published within the journal article, if accepted, and that failure to comply and/or to accurately and completely report the potential financial conflicts of interest could lead to the following: 1) Prior to publication, article rejection, or 2) Post-publication, sanctions ranging from, but not limited to, issuing a correction, reporting the inaccurate information to the authors' institution, banning authors from submitting work to ASN journals for varying lengths of time, and/or retraction of the published work.

Name: Juliano Alhaddad
Manuscript ID: K360-2026-000992
Manuscript Title: Nephrology's Onboarding Challenge may reflect a Workforce Challenge
Date of Completion: August 19, 2026
Disclosure Updated Date: August 19, 2026

ASN American Society of Nephrology
JASN Journal of the American Society of Nephrology
KIDNEY360 Accessing Our World From Every Angle
CJASN Clinical Journal of the American Society of Nephrology

ASN Journal Disclosure Form

As per ASN journal policy, I have disclosed any financial relationships or commitments I have held in the past 36 months as included below. I have listed my Current Employer below to indicate there is a relationship requiring disclosure. If no relationship exists, my Current Employer is not listed.

G. Nakhoul reports the following:
Employer: Cleveland Clinic Foundation; Consultancy: Amgen, Novo Nordisk, Travere therapeutics; and Speakers Bureau: Amgen, Novo Nordisk.

I understand that the information above will be published within the journal article, if accepted, and that failure to comply and/or to accurately and completely report the potential financial conflicts of interest could lead to the following: 1) Prior to publication, article rejection, or 2) Post-publication, sanctions ranging from, but not limited to, issuing a correction, reporting the inaccurate information to the authors' institution, banning authors from submitting work to ASN journals for varying lengths of time, and/or retraction of the published work.

Name: Georges Nakhoul
Manuscript ID: K360-2026-000992
Manuscript Title: Nephrology's Onboarding Challenge may reflect a Workforce Challenge
Date of Completion: August 13, 2026
Disclosure Updated Date: August 13, 2026
